# Impact of CD44 genetic variants on clinicopathological characteristics of uterine cervical cancer patients

**DOI:** 10.7150/ijms.96414

**Published:** 2024-05-27

**Authors:** Yi-Hung Sun, Ching-Ming Wang, Huang-Pin Shen, Chung-Yuan Lee, Chiao-Wen Lin, Shun-Fa Yang, Po-Hui Wang

**Affiliations:** 1 Department of Obstetrics and Gynecology, Chi-Mei Foundation Medical Center, Tainan, Taiwan.; 2 School of Medicine, Kaohsiung Medical University, Kaohsiung, Taiwan.; 3 Institute of Medicine, Chung Shan Medical University, Taichung, Taiwan.; 4 School of Medicine, National Defense Medical Center, Taipei, Taiwan.; 5 School of Medicine, College of Medicine, National Cheng Kung University, Tainan, Taiwan.; 6 School of Medicine, Chung Shan Medical University, Taichung, Taiwan.; 7 Department of Obstetrics and Gynecology, Chung Shan Medical University Hospital, Taichung, Taiwan.; 8 Department of Obstetrics and Gynecology, Chiayi Chang Gung Memorial Hospital Chiayi, Taiwan.; 9 Department of Nursing, Chang Gung University of Science and Technology, Chiayi Campus, Chiayi, Taiwan.; 10 Institute of Oral Sciences, Chung Shan Medical University, Taichung, Taiwan.; 11 Department of Medical Research, Chung Shan Medical University Hospital, Taichung, Taiwan.

**Keywords:** CD44, genetic variants, rs187115, cancer of uterine cervix, 5 years survival rate

## Abstract

CD44 genetic variants have been found to be related to various cancers. However, to date, no study has demonstrated the involvement of CD44 polymorphisms in uterine cervical cancer in Taiwanese women. Therefore, we conducted a retrospective study, consecutively recruiting 113 patients with invasive cancer, 92 patients with high-grade cervical intraepithelial neoplasias, and 302 control women to assess the relationships among CD44 polymorphisms, cervical carcinogenesis, and patient survival. Real-time polymerase chain reaction was used to determine the genotypic distributions of six polymorphisms: rs1425802, rs187115, rs713330, rs11821102, rs10836347, and rs13347. The results revealed that women with the mutant homozygous genotype CC exhibited a higher risk of invasive cancer compared to those with the wild homozygous genotype TT [p=0.035; hazard ratio (HR)=10.29, 95% confidence interval (95% CI)=1.18-89.40] and TT/TC [p=0.032; HR=10.66, 95% CI=1.23-92.11] in the CD44 polymorphism rs713330. No significant association was found between CD44 genetic variants and clinicopathological parameters. Among the clinicopathological parameters, only positive pelvic lymph node metastasis (p=0.002; HR=8.57, 95% CI=2.14-34.38) and the AG/GG genotype compared to AA (p=0.014; HR=3.30, 95% CI=1.28-8.49) in CD44 polymorphism rs187115 predicted a higher risk of poor five-year survival, according to multivariate analysis. In conclusion, an important and novel finding revealed that Taiwanese women with the AG/GG genotype in CD44 polymorphism rs187115 exhibited a higher risk of poor five-year survival.

## Introduction

Cancer of the uterine cervix ranked as the fourth most frequent cancer in female subjects, with approximately 604,127 women newly diagnosed and 341,831 deaths worldwide in 2020 1. However, in Taiwan, the annual age-standardized incidence rate of cervical cancer was estimated to be 7.04 per 100,000 women, ranking as the eleventh most common cancer among women in 2021, according to the Health Promotion Administration of the Ministry of Health and Welfare and the Annual Cancer Registry Report. The mortality rate was the eighth leading cause of cancer mortality among Taiwanese women, calculated to be 2.75 per 100,000 women.

Cervical carcinogenesis is regarded as a continuous, multi-step process of neoplastic transformation, from cervical intraepithelial neoplasia (CIN) considered as precancerous lesions, to invasive cancer known as the final step of CIN progression 2-4. When mitoses and immature cells account for the lower one-third of cervical epithelium, the cytological term low-grade squamous cell intraepithelial lesions is defined, and the histological term CIN 1 is used (also known as low-grade CIN or dysplasia, or mild dysplasia). If mitoses and immature cells occupy the middle and upper third of the epithelium, they are histologically referred to as CIN 2 (moderate dysplasia) and CIN 3 (severe dysplasia and carcinoma in situ when the whole epithelium is occupied), respectively. Collectively, these are regarded as high-grade CIN or high-grade dysplasia and known as precancerous lesions. Their cytological counterpart is termed high-grade squamous cell intraepithelial lesions 5.

In humans, the CD44 gene is located on chromosome 11p13, consisting of 20 exons, including 10 constant exons and 10 variant exons 6, 7. The surface glycoproteins CD44 are members of the hyaluronate receptor family and are known as major adhesion molecules of the extracellular matrix 8. In addition to mediating cellular adhesion to the cell-extracellular matrix, CD44 plays important roles in the differentiation, invasion, and metastasis of tumor cells 9-11. If a different allele exists in the shared DNA sequence of a gene between members of a species or paired chromosomes with a frequency of more than 5% in a certain population, a single nucleotide polymorphism (SNP) occurs 12, 13. The genetic variant can impact gene expression by influencing the promoter area, exon, or 3'-untranslated region, leading to genetic susceptibilities and subsequently affecting the occurrence of diseases and cancers 14, 15.

It has been reported that CD44 polymorphisms are associated with susceptibility to different cancers 16-19. However, no report has investigated the association between CD44 genetic variants and uterine cervical cancer in Taiwan. Therefore, we designed this research to relate CD44 genetic polymorphisms to cervical carcinogenesis, as well as clinicopathological parameters and the 5-year survival rate of Taiwanese cervical cancer patients.

## Materials and methods

### Enrolled population

A retrospective study was conducted to consecutively recruit 113 patients with invasive cancer and 92 patients with high-grade cervical intraepithelial neoplasias (high-grade CINs, precancerous lesions) from the Department of Obstetrics and Gynecology affiliated with Chung Shan Medical University Hospital in Taichung, Taiwan, from February 1994 to February 2015. Simultaneously, 302 women who had never been diagnosed with CINs and who received routine examinations in the outpatient department of the hospital were regarded as the control group. These participants were considered control women if they had normal cytologic reports from cervical Papanicolaou smears and were further confirmed by normal colposcopic findings during general examinations. All individuals were Taiwanese residents of central Taiwan. The marital status and education level were comparable between cases and controls. Colposcopy-directed cervical biopsies were performed, and pathological reports verified the diagnoses of invasive cancer and high-grade CINs. Patients with invasive cervical cancer and precancerous lesions were classified as patients with cervical neoplasias. These patients received the standard treatment protocols as revised by the hospital, which were based on the guidelines of the National Comprehensive Cancer Network. The Institutional Review Board of the Affiliated Hospital of Chung Shan Medical University supervised the study (CSMUH number: CS18208). Informed consent was obtained from all subjects.

### Definition of CD44 genetic variants

Six CD44 genetic variants were checked based on the data of International HapMap Project and previous investigations 16, 20. Jiang *et al.* revealed that the CD44 single nucleotide polymorphism (SNP) rs1425802 locates in the promoter region. Moreover, rs11821102, rs10836347 and rs13347 situate in the 3'UTR area, and they have been showed to influence the binding function of certain MicroRNA in a Chinese population 16. SNPs rs187115 and rs713330 were other 2 common studied CD44 variants 21, 22.

### Extraction of deoxyribonucleic acid (DNA) from all subjects' blood samples and real time polymerase chain reaction

Laboratory staff used venipuncture techniques to draw blood specimens from all subjects. The specimens were put into Vacutainer tubes mixed with ethylenediaminetetraacetic acid, and immediately stored at 4 °C. Genomic DNA was subsequently extracted from leukocytes by QIAamp DNA blood mini kits in accordance with manufacturer's instructions as previous study 23, 24. Extracted DNA was further dissolved in pH 7.8 TE buffer (10 mM Tris and 1 mM EDTA; pH 7.8). Thereafter, it was detected by the measurement of optical density at OD260. The OD260/OD280 ratio was defined and the range of 1.8-2.0 accorded with our criteria. The product was considered as pure and its cross reactivity with the current homologous RNA was prevented. Then, the final products were refrigerated at -20 °C and regarded as templates for the polymerase chain reaction (PCR).

The allelic determination of 6 CD44 genetic variants, rs1425802 (assay ID: C_7618925_20), rs187115 (assay ID: C_779820_10), rs713330 (assay ID: C_779798_20), rs11821102 (assay ID: C_2143187_10), rs10836347 (assay ID: C_32034604_10), and rs13347 (assay ID: C_7619022_10) were detected by ABI StepOne Real-Time PCR System (Applied Biosystems, Foster City, CA, USA) as well as defined by SDS version 3.0 software (Applied Biosystems) with the TaqMan assay 25.

### Statistical analysis

Analysis of variance (ANOVA) was applied for the comparison of the age distribution of the participants using the Brown-Forsythe test, and the Games-Howell test for post hoc analysis. Chi-squared and Fisher's exact tests were performed to associate the genotypic frequencies of six CD44 genetic polymorphisms with the incidence of cervical neoplasias. Age adjustment was necessary because the age of patients suffering from invasive cancer was older than that of patients with precancerous lesions of the uterine cervix. The p-values, odds ratios (ORs), and adjusted ORs (AORs) with their 95% confidence intervals (95% CIs) were calculated or adjusted for age using chi-squared and Fisher's exact tests, or logistic and multinomial logistic regression models, and used to assess the involvement of CD44 polymorphisms in cervical carcinogenesis. Chi-squared or Fisher's exact tests were applied to relate CD44 genetic polymorphisms to clinicopathological factors of cervical cancer patients. The associations between death events and CD44 variants, as well as clinicopathological parameters, were assessed by the p-values using chi-squared or Fisher's exact tests. The Kaplan-Meier curve model (univariate analysis over time) was used to define the prognostic prediction of CD44 polymorphisms and clinicopathological characteristics for the 5-year survival rates in patients with invasive cervical cancer. Differences were defined by the log-rank test. The impacts of CD44 genetic variants and the clinicopathological parameters on the 5-year survival of these patients were assessed using the Cox proportional hazard model for multivariate analysis in relation to survival time. The hazard ratios (HRs) were then determined. SPSS, version 18.0, and WinPepi Software, version 10.0, were used for statistical analysis.

## Results

### Age distribution of studied population

There was a significant difference for the age distribution between patients with cervical neoplasm and control females (50.4 ± 13.8 vs. 44.0 ± 10.0, *p*<0.001) for the Taiwanese population. There was a significant difference among patients with invasive cancer and precancerous lesions of uterine cervix as well as control women based on the Brown-Forsythe test (*p*<0.001). Using Games-Howell post hoc analysis, the age differences were significant between patients with cervical cancer and patients with precancerous lesions (55.7 ± 12.6 vs. 43.7 ± 12.3, *p*<0.001) as well as between cervical cancer patients and control women (55.7 ± 12.6 vs. 44.0 ± 10.0, *p*<0.001). But, no significant difference was noted for the age distribution between patients with precancerous lesions and control females (43.7 ± 12.3 vs. 44.0 ± 10.0, *p*= 0.988).

### Involvement of CD44 genetic variants in carcinogenesis of uterine cervix

The minor allele frequencies of CD44 genetic variants rs1425802, rs187115, rs713330, rs11821102, rs10836347 and rs13347 were all ≥5%. The CD44 genetic variants in the Taiwanese women with neoplasias of uterine cervix and control women are presented in Table 1. A significant difference only existed in the distribution of CD44 genetic variants rs11821102 among the 6 polymorphisms between women with cervical neoplasias and control women (*p*=0.039). Individuals with genotype GA in rs11821102 had more risk of developing cervical neoplasias, as compared to those with G/G. However, after adjusting for age, the significant difference did not reach.

Cervical neoplasias group was further categorized into subgroups of precancerous lesions and invasive cancer, and then CD44 genetic variants were assessed to their relationships with cervical carcinogenesis. There were significantly different genotype distributions of TT, TC and CC (*p*=0.026) as well as of TT and TC & CC (*p*=0.025) in CD44 polymorphism rs713330 among patients with invasive cancer and precancerous lesions as well as control women. After age adjustment, women with heterozygous genotype AG & mutant homozygous genotype GG exhibited more risk to have cervical precancerous lesions as compared to those with wild homozygous genotype AA in rs187115 (*p*=0.046; AOR=1.62, 95% CI=1.01-2.59; Table 2). Individuals with heterozygous genotype TC had more risk of developing precancerous lesions as compared with those with wild homozygous genotype TT (*p*=0.009; AOR=2.15, 95% CI=1.21-3.84; Table 2) in the comparison of genotypes TT, TC and CC and women with heterozygous genotype TC & mutant homozygous genotype CC presented more risk of developing precancerous lesions as compared with those with TT (*p*=0.014; AOR=2.05, 95% CI=1.15-3.63; Table 2) in CD44 polymorphism rs713330. Women with genotype GA presented more risk of developing precancerous lesions as compared with those with GG (*p*=0.044; AOR=1.93, 95% CI=1.01-3.68; Table 2) in the comparison of genotypes GG, GA and AA in rs11821102. Women with genotype CC exhibited more risk to have invasive cancer as compared with those with TT (*p*=0.035; AOR=10.29, 95% CI=1.18-89.40; Table 2) in the comparison of genotypes TT, TC and CC and women with CC presented more risk of developing precancerous lesions as compared with those with TT/TC (*p*=0.032; AOR=10.66, 95% CI=1.23-92.11; Table 2) in CD44 polymorphism rs713330.

### Associations between CD44 polymorphisms and clinicopathological parameters of cervical cancer patients

Moreover, the relationships between CD44 genetic variants and clinicopathological parameters of cervical cancer patients were assessed. Only patients with genotypes GA/AA in rs11821102 had the tendency of vagina invasion as compared to those with GG (Table 4). Otherwise, no CD44 genetic polymorphisms displayed significant relationships with these parameters.

### Clinical implication of CD44 genetic variants in patient prognosis

**Figure 1:**
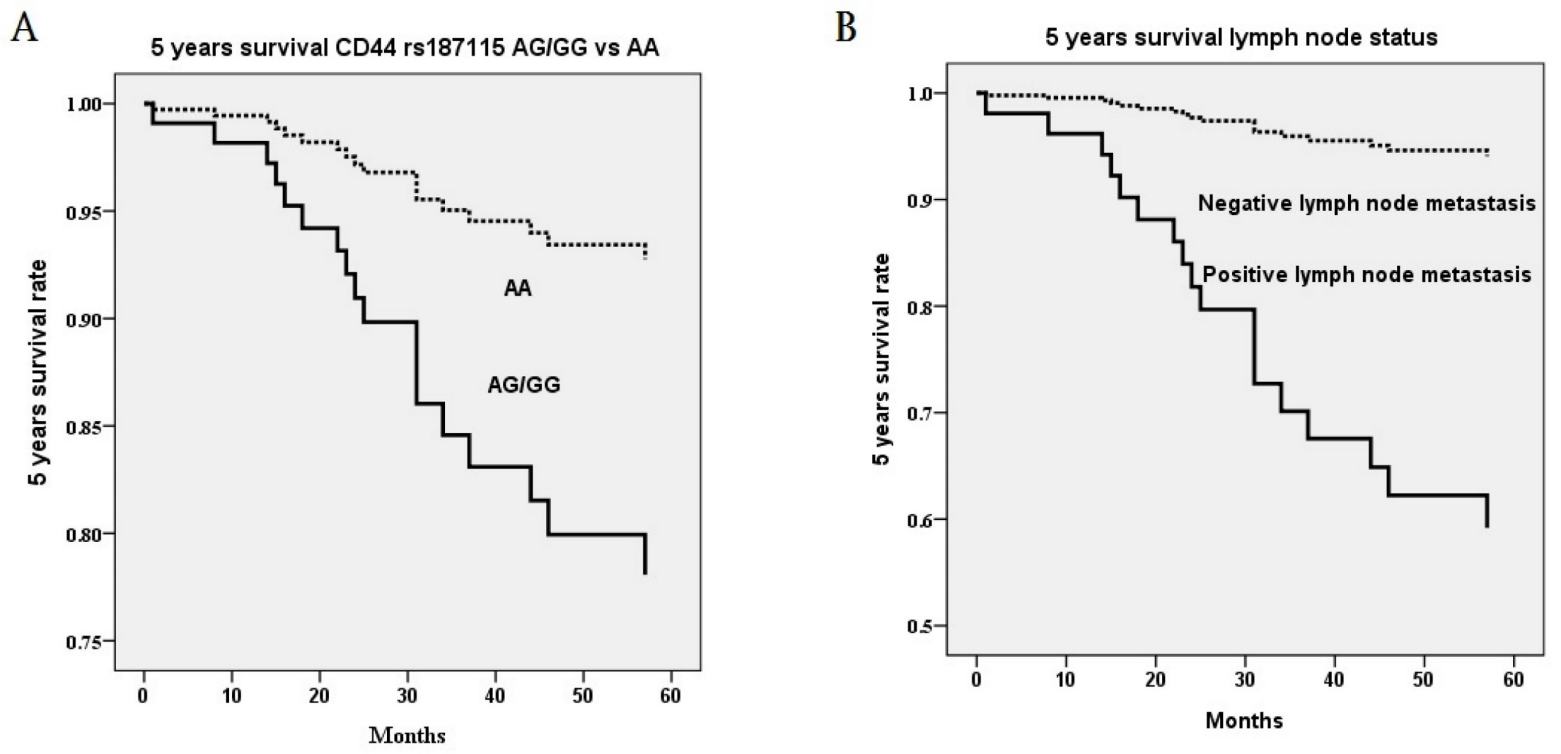
Five years survival rate based on the only significant polymorphism rs187115 among CD44 genetic variants and only significant clinicopathological parameter pelvic lymph node metastasis in multivariate Cox proportional hazard model. (A) genotypes AG/GG exhibit more risk of poor 5 years survival rate as compared to genotype AA (*p*=0.014; hazard ratio: 3.30, 95% confidence: 1.28-8.49) (B) positive pelvic lymph node metastasis exhibits more risk of poor 5 years survival rate as compared to negative pelvic lymph node metastasis (*p*=0.002; hazard ratio: 8.57, 95% confidence: 2.14-34.38). Statistical analysis: Cox proportional hazard model after adjusting for various CD44 genetic variants and clinicopathological parameters.

Noteworthily, CD44 genetic variants were related to 5 years survival of cervical cancer patients. In univariate analysis, the death event was associated with heterozygous genotype AG and mutant homozygous genotype GG as compared to wild homozygous genotype AA in CD44 polymorphism rs187115 (*p*=0.026), ≥ stage II as compared to stage I (*p*=0.005), > 10 mm as compared to ≤ 10 mm stromal invasion depth (*p*=0.003), > 4 cm as compared to ≤ 4 cm tumor diameter (*p*=0.004), positive as compared to negative parameter invasion (*p*=0.003), and positive as compared to negative pelvic lymph node metastasis (*p*<0.001; Table 4). When survival time interval was included for 5-year analysis, patients with genotypes AG/GG exhibited more risk of poor 5-year survival rate (HR=2.67, 95% CI=1.08-6.59; Table 4). Cervical patients with > stage II (HR=3.47, 95% CI=1.33-9.04), deep stromal invasive (HR=4.56, 95% CI=1.51-135.7), large tumor diameter (HR=4.03, 95% CI=1.46-11.09), positive parametrium invasion (HR=3.42, 95% CI=1.36-8.57) and positive lymph node metastasis (HR=10.04, 95% CI=3.46-27.70) also exerted more risk of 5-year survival rate (Table 4)**.** However, in multivariate analysis, only patients with AG/GG as compared to AA (*p*=0.014; HR=3.30, 95% CI=1.28-8.49; Figure 1A) in CD44 polymorphism rs187115 and those with positive pelvic lymph node metastasis (*p*=0.002; HR=8.57, 95% CI=2.14-34.38) had more risk of poor 5-year survival (Table 5; Figure 1B).

## Discussion

This study revealed that Taiwanese women with the heterozygous genotype GA had a higher risk of suffering from cervical neoplasias compared to those with the wild homozygous genotype GG in the comparison of GG, GA, and AA in rs11821102 among six CD44 genetic variants. However, after adjusting for age, the significant difference disappeared. Notably, patients with cervical neoplasias did not have the AA genotype in the investigation. After subdividing cervical neoplasias into precancerous lesions and invasive cancer and adjusting for age, Taiwanese women with genotypes AG/GG compared to AA in CD44 genetic variant rs187115, women with GA compared to GG in rs11821102, and those carrying the mutant allele C compared to allele T in rs713330 had a higher risk of suffering from cervical precancerous lesions. Additionally, women with the mutant homozygous genotype CC had a higher risk of progressing to invasive cervical cancer compared to those with the wild homozygous genotype TT in rs713330. To our knowledge, this is the first study to investigate the involvement of CD44 SNPs in cervical carcinogenesis. In contrast, Chou *et al.* revealed that subjects with genotypes AG/GG had a higher risk of developing hepatocellular carcinoma compared to AA in rs187115 in Taiwan 26. Furthermore, Taiwanese individuals with AG/GG had a higher risk of having transitional cell carcinoma of the urinary bladder compared to AA in rs187115 17. Conversely, Chen *et al.* indicated that rs187115 polymorphism was related to the risk of lung and liver diseases but not to the risk of breast, gastric, colon, or rectal cancer in a central Chinese population 27.

CD44 polymorphism rs713330 has been shown to correlate with clinicopathological characteristics in other cancers. However, in this study, no significant associations between CD44 genetic polymorphisms and clinicopathological parameters were found. In contrast, it has been demonstrated that male patients with lung adenocarcinoma who have the TC genotype in rs713330 exhibit significant relationships with tumor size and invasion, particularly in patients presenting with the wild-type epidermal growth factor receptor in Taiwan 20. Rs713330 is located in the intron of CD44 and is associated with the disequilibrium of the nonsynonymous rs9666607 GA polymorphism, which results in the change of arginine to lysine at residue 417. This change likely affects the subtype and content of CD44 mRNA and/or protein, thus impacting clinicopathological characteristics. CD44 has been reported to be involved in tumor invasion, metastasis, and epithelial to mesenchymal transition 10, 28, 29.

This study presented an important and unique finding. In addition to univariate Kaplan-Meier curve model analysis, multivariate Cox proportional hazard analysis also revealed that cervical cancer patients with genotypes AG/GG had a higher risk of poor 5-year survival rates compared to AA in CD44 polymorphism rs187115. This significant finding was also observed in patients with positive pelvic lymph node metastasis. CD44 genetic variant rs187115 is located in the first intron of CD44. Although no regulatory role for intron 1 of CD44 has been found, a similar intron 1 CD44 polymorphism was demonstrated to be associated with altered splicing of CD44 and affect its expression in breast cancer by Zhou et al 30. Furthermore, elevated expression of CD44 has been found to be associated with decreased survival in patients with oral squamous cell carcinoma 31-33. Additionally, Stracquadanio *et al.* reported that CD44 genetic variant rs187115 could be identified as a diagnostic biomarker for pancreatic ductal adenocarcinoma and was associated with tumor progression 21. Vazquez *et al.* showed that patients with soft sarcoma carrying genotype GG in CD44 polymorphism rs187115 exhibited poorer overall survival as compared to those with genotypes AG/AA 34. Moreover, Wan *et al.* found that patients with colorectal cancer who carried the mutant homozygous genotype GG exhibited poorer overall survival than those with AA in a Chinese Han population 35. However, Jiang *et al.* demonstrated that genotypes CT/TT in CD44 rs13347 predicted poorer 5-year survival rates for breast cancer patients in a Chinese population 16.

This study has an important and novel finding: Taiwanese women with cervical cancer who carry genotypes GG/AG in CD44 genetic variant rs187115 have poorer 5-year survival rates than those with AA. However, some limitations exist. First, the study design was a retrospective hospital-based cohort study, which may introduce selection bias. Nevertheless, in this study, cases and controls were enrolled from the same hospital, and cervical cancer patients whose blood samples were collected in the same hospital were sporadic, thus minimizing the probability of selection bias. Second, this study only recruited participants from central Taiwan and did not include women from other regions. Subjects were enrolled only if all their six CD44 genetic variants could be defined. The sample size might not be large enough to reach significant differences, particularly in the precancerous lesions group, thus limiting possible subgroup analysis and affecting external validity. Third, the ages at which patients with cervical precancerous lesions and those with invasive cancer occur are inherently different, leading to different age distributions for these diseases. Therefore, logistic regression models with age adjustment were used to reduce the impact of age. Fourth, female subjects in the control group were included from the outpatient clinic of Chung Shan Medical University Hospital for general examinations. Due to the conservative attitude of Taiwanese women, examination for human papillomavirus (HPV) infection was not performed routinely. Consequently, the influence of HPV could not be included in the analysis.

## Figures and Tables

**Table 1:** Genetic variant frequencies of CD44 in Taiwanese females with cervical neoplasias and normal controls

Genetic variants	Normal controls (n = 302)	Cervical neoplasias^a^ (n= 205)	ORs (95% CIs)	*p* values	AORs (95% CIs)^b^	Adjusted *p* values^b^
**rs1425802**						
AA^c^	99	55	1.00	0.292	1.00	0.277
AG	155	110	1.28 (0.85-1.93)	0.242	1.31 (0.86-2.01)	0.210
GG	48	40	1.50 (0.88-2.56)	0.136	1.53 (0.87-2.67)	0.137
AA^c^	99	55	1.00		1.00	
AG & GG	203	150	1.33 (0.90-1.97)	0.153	1.36 (0.91-2.05)	0.136
AA & AG^c^	254	165	1.00		1.00	
G/G	48	40	1.28 (0.81-2.04)	0.292	1.28 (0.79-2.08)	0.315
**rs187115**						
AA^c^	199	126	1.00	0.592	1.00	0.588
AG	94	66	1.11 (0.75-1.63)	0.599	1.18 (0.79-1.77)	0.416
GG	14	13	1.47 (0.67-3.22)	0.340	1.37 (0.61-3.07)	0.449
AA^c^	199	126	1.00		1.00	
AG & GG	108	79	1.16 (0.80-1.67)	0.440	1.21 (0.83-1.77)	0.331
AA & AG^c^	293	192	1.00		1.00	
GG	14	13	1.42 (0.65-3.08)	0.379	1.29 (0.58-2.87)	0.527
**rs713330**						
TT^c^	260	168	1.00	0.434	1.00	0.397
TC	41	36	1.36 (0.83-2.21)	0.218	1.29 (0.78-2.15)	0.323
CC	2	2	1.55 (0.22-11.09)	0.664	2.72 (0.36-20.58)	0.331
TT^c^	260	168	1.00		1.00	
TC & CC	43	38	1.37 (0.85-2.21)	0.199	1.34 (0.82-2.21)	0.248
TT & TC^c^	301	204	1.00		1.00	
CC	2	2	1.48 (0.21-10.56)	0.698	2.63 (0.35-19.81)	0.349
**rs11821102**						
GG^c^	268	170	1.00	0.118	1.00	0.200
GA	32	35	1.72 (1.03-2.89)	0.039^*^	1.63 (0.96-2.78)	0.073
AA	4	0	u.a.	0.999	u.a.	0.999
GG^c^	268	170	1.00		1.00	
GA & AA	36	35	1.53 (0.93-2.54)	0.096	1.46 (0.87-2.45)	0.157
GG & GA^c^	300	205	1.00		1.00	
A/A	4	0	u.a.	0.999	u.a.	0.999
**rs10836347**						
CC^c^	263	173	1.00	0.540	1.00	0.753
CT	41	32	1.19 (0.72-1.96)	0.503	1.19 (0.71-2.00)	0.514
TT	1	2	3.04 (0.27-33.79)	0.365	1.63 (0.14-18.72)	0.694
CC^c^	263	173	1.00		1.00	
CT & TT	42	34	1.23 (0.75-2.01)	0.408	1.20 (0.72-2.01)	0.476
CC & CT^c^	304	205	1.00		1.00	
TT	1	2	2.97 (0.27-32.92)	0.376	1.59 (0.14-18.25)	0.708
**rs13347**						
CC^c^	145	94	1.00	0.920	1.00	0.839
CT	130	91	1.08 (0.74-1.57)	0.687	1.11 (0.75-1.64)	0.595
TT	38	26	1.06 (0.60-1.85)	0.851	0.98 (0.55-1.75)	0.937
CC^c^	145	94	1.00		1.00	
CT & TT	168	117	1.07 (0.76-1.53)	0.689	1.08 (0.75-1.55)	0.684
CC & CT^c^	275	185	1.00		1.00	
TT	38	26	1.02 (0.60-1.73)	0.950	0.93 (0.54-1.61)	0.795

Statistical analysis: logistic regression model or chi-square or Fisher's tests. ^a^Cervical neoplasias consist of precancerous lesions and invasive cancer of the uterine cervix. ^b^The adjusted *p* values as well as adjusted odds ratios (AORs) and their 95% confident intervals (95% CIs) were calculated by logistic regression model after age adjustment. ^c^Used as a reference for comparison to assess the odds ratios of other genotypes. ^*^p<0.05

**Table 2:** Genetic variant frequencies of CD44 in Taiwanese females with uterine cervical invasive cancer or precancerous lesion and normal controls

Genetic variants	Normal controls (n =302)	Precancerous lesions (n =92)	Invasive cancer (n =113)	*p* values	AORs (95% CIs)^a^	Ad. *p* values	AORs (95% CIs)^b^	Ad. *p* values
**rs1425802**								
AA^c^	99	26	29	0.610	1.00		1.00	
AG	155	49	61		1.20 (0.70-2.06)	0.499	1.49 (0.85-2.61)	0.169
GG	48	17	23		1.35 (0.67-2.72)	0.403	1.71 (0.83-3.52)	0.145
AA^c^	99	26	29	0.332	1.00		1.00	
AG & GG	203	66	84		1.24 (0.74-2.07)	0.415	1.54 (0.90-2.64)	0.117
AA & AG^c^	254	75	90	0.538	1.00		1.00	
GG	48	17	23		1.20 (0.65-2.21)	0.558	1.32 (0.71-2.45)	0.373
**rs187115**								
AA^c^	199	49	77	0.191	1.00		1.00	
AG	94	36	30		1.56 (0.95-2.55)	0.081	0.86 (0.50-1.48)	0.592
GG	14	7	6		2.03 (0.78-5.32)	0.149	0.98 (0.34-2.87)	0.977
AA^c^	199	49	77	0.066	1.00		1.00	
AG & GG	108	43	36		1.62 (1.01-2.59)	0.046^*^	0.88 (0.53-1.46)	0.621
AA & AG^c^	293	85	107	0.466	1.00		1.00	
GG	14	7	6		1.73 (0.67-4.43)	0.255	1.03 (0.36-2.96)	0.960
**rs713330**								
TT^c^	260	68	100	0.026^*^	1.00		1.00	
TC	41	23	13		2.15 (1.21-3.84)	0.009^*^	0.62 (0.29-1.30)	0.207
CC	2	0	2		u.a.	u.a.	10.29 (1.18-89.40)	0.035^*^
TT^c^	260	68	100	0.025^*^	1.00		1.00	
TC & CC	43	23	15		2.05 (1.15-3.63)	0.014^*^	0.75 (0.37-1.51)	0.420
TT & TC^c^	301	91	113	0.360	1.00		1.00	
CC	2	0	2		u.a.	u.a.	10.66 (1.23-92.11)	0.032^*^
**rs11821102**								
GG^c^	268	74	96	0.137	1.00		1.00	
GA	32	17	18		1.93 (1.01-3.68)	0.044^*^	1.39 (0.70-2.76)	0.342
AA	4	0	0		u.a.	u.a.	u.a.	u.a.
GG^c^	268	74	96	0.208	1.00		1.00	
GA & AA	36	17	18		1.72 (0.91-3.23)	0.094	1.25 (0.64-2.44)	0.516
GG & GA^c^	300	91	114	0.483	1.00		1.00	
A/A	4	0	0		u.a.	u.a.	u.a.	u.a.
**rs10836347**								
CC^c^	263	81	92	0.356	1.00		1.00	
CT	41	11	21		0.87 (0.43-1.77)	0.697	1.58 (0.83-3.02)	0.169
TT	1	1	1		3.42 (0.21-56.50)	0.390	0.98 (0.06-17.40)	0.987
CC^c^	263	81	92	0.310	1.00		1.00	
CT & TT	42	12	22		0.93 (0.47-1.85)	0.829	1.52 (0.80-2.87)	0.200
CC & CT^c^	304	92	113	0.358	1.00		1.00	
TT	1	1	1		3.48 (0.21-57.35)	0.384	0.92 (0.05-16.28)	0.952
**rs13347**								
CC^c^	145	45	49	0.576	1.00		1.00	
CT	130	35	56		0.86 (0.52-1.43)	0.564	1.35 (0.82-2.21)	0.242
TT	38	14	12		1.19 (0.59-2.40)	0.620	0.77 (0.35-1.71)	0.527
CC^c^	145	45	49	0.633	1.00		1.00	
CT & TT	168	49	68		0.94 (0.59-1.49)	0.785	1.19 (0.75-1.91)	0.464
CC & CT^c^	275	80	105	0.592	1.00		1.00	
TT	38	14	12		1.28 (0.66-2.48)	0.473	0.67 (0.32-1.43)	0.301

^a^Adjusted *p* values and adjusted odds ratios with their 95% CIs were calculated using multinomial logistic regression models after age adjustment between patients with uterine cervical precancerous lesions and control females. ^b^Adjusted *p* values and adjusted odds ratios with their 95% CIs were calculated using multinomial logistic regression models after age adjustment between patients with uterine cervical invasive cancer and control females. ^c^Used as a reference for comparison to assess the odds ratios of other genotypes. AORs, adjusted odds ratios; 95% CIs, 95% confidence intervals; Ad. *p*, adjusted *p*. ^*^p<0.05

**Table 3:** Relationships between genotypic distributions of CD44 and clinicopathological parameters of the patients with cervical invasive cancer.

**Parameters^a^**	**rs1425802**				**rs187115**				**rs713330**			
	**AA^b^**	**AG/GG**	**AA/AG^b^**	**GG**	**AA^b^**	**AG/GG**	**AA/AG^b^**	**GG**	**TT^b^**	**TC/CC**	**TT/TC^b^**	**CC**
**Clinical stage**												
stage I^b^	15	50	51	14	41	24	62	3	58	9	66	1
≥stage II	14	33	38	9	36	11	45	2	41	6	46	1
* p* value	0.424		0.757		0.128		1.000		0.917		1.000	
**Pathologic type**												
squamous cell carcinoma^b^	24	76	78	22					88	13	99	2
adenocarcinoma	5	7	11	1					11	2	13	0
* p* value	0.292		0.454		0.335		1.000		0.680		1.000	
**Cell grading**												
well (grade 1)^b^	4	14	15	3	15	3	18	0	17	1	17	1
moderate & poor (grades 2/3)	25	69	74	20	62	32	89	5	82	14	95	1
* p* value	1.000		1.000		0.145		1.000		0.459		0.292	
**Stromal invasion depth**												
≤10 mm^b^	13	46	46	13	37	22	55	4	54	6	59	1
>10 mm	15	35	40	10	37	13	49	1	42	9	50	3
* p* value	0.343		0.795		0.208		0.372		0.240		1.000	
**Tumor diameter**												
≤4 cm^b^	14	47	48	13	39	22	57	4	55	7	61	1
>4 cm	14	36	40	10	37	13	49	1	43	8	50	1
* p* value	0.542		0.865		0.256		0.376		0.493		1.000	
**Parametrium**												
no invasion^b^	16	55	57	14	47	24	66	5	62	11	71	2
invasion	12	28	31	9	29	11	40	0	36	4	40	0
* p* value	0.385		0.728		0.493		0.157		0.448		0.539	
**Vagina**												
no invasion^b^	14	55	56	13	43	26	64	5	61	10	70	1
invasion	14	28	32	10	33	9	42	0	37	5	41	1
* p* value	0.125		0.531		0.074		0.155		0.741		1.000	
**Pelvic lymph node**												
no metastasis^b^	19	62	63	18	55	26	76	5	72	11	81	2
metastasis	9	21	25	5	21	9	30	0	26	4	30	0
* p* value	0.481		0.521		0.833		0.321		1.000		1.000	
**Parameters^a^**	**rs11821102**				**rs10836347**				**rs13347**			
	**GG^b^**	**GA/AA**	**GG/GA^b^**	**AA**	**CC^b^**	**CT/TT**	**CC/CT^b^**	**TT**	**CC^b^**	**CT/TT**	**CC/CT^b^**	**TT**
**Clinical stage**												
stage I^b^	58	8	66	0	54	11	65	0	28	40	58	10
≥stage II	38	9	47	0	38	10	47	1	20	28	46	2
* p* value	0.303		u.a.		0.597		0.425		0.958		0.119	
**Pathologic type**												
squamous cell carcinoma^b^	84	17	101	0	80	20	99	1	41	63	93	11
adenocarcinoma	12	0	12	0	12	1	13	0	7	5	11	1
* p* value	0.208		u.a.		0.456		1.000		0.230		1.000	
**Cell grading**												
well (grade 1)^b^	18	0	18	0	13	5	18	0	8	10	18	0
moderate & poor (grades 2/3)	78	17	95	0	79	16	94	1	40	58	86	12
* p* value	0.069		u.a.		0.322		1.000		0.774		0.209	
**Stromal invasion depth**												
≤10 mm^b^	51	8	59	0	51	8	59	0	26	34	53	7
>10 mm	42	9	51	0	38	13	50	1	22	31	48	5
* p* value	0.554		u.a.		0.112		0.464		0.845		0.701	
**Tumor diameter**												
≤4 cm^b^	53	8	61	0	50	12	62	0	28	35	55	8
>4 cm	42	9	51	0	41	9	49	1	20	32	48	4
* p* value	0.506		u.a.		0.855		0.446		0.517		0.382	
**Parametrium**												
no invasion^b^	64	8	72	0	60	12	72	0	31	43	63	11
invasion	31	9	40	0	31	9	39	1	17	24	40	1
* p* value	0.107		u.a.		0.449		0.357		0.964		0.053	
**Vagina**												
no invasion^b^	63	7	70	0	58	12	70	0	28	45	62	11
invasion	32	10	42	0	33	9	41	1	20	22	41	1
* p* value	0.049^*^		u.a.		0.574		0.375		0.332		0.053	
**Pelvic lymph node**												
no metastasis^b^	72	10	82	0	67	14	81	0	35	49	74	10
metastasis	23	7	30	0	24	7	30	1	13	18	29	2
* p* value	0.232		u.a.		0.521		0.277		0.979		0.509	

Statistical analyses: chi-square or Fisher's exact tests ^*^p<0.05 ^a^Clinicopathological data of some cases could not be obtained from the patients with cervical invasive cancer because of incomplete medical charts or records. ^b^As a reference.

**Table 4:** Univariate analysis of genetic variants of CD44 and clinicopathological variables for 5-year survival in cervical cancer patients

	5-year survival event^c^			5-year survival rate^d^
Parameters^a^	+	-	*p* value	HR (95% CIs)^c^
**CD44 genetic polymorphisms**				
**rs1425802**				
AA^b^	24	4	0.776	1.00
AG/GG	66	15		1.14 (0.38-3.45)
AA/AG^b^	72	15	1.000	1.00
GG	18	4		1.04 (0.34-3.13)
**rs187115**				
AA^b^	66	9	0.026^*^	1.00
AG/GG	24	10		2.67 (1.08-6.59)
AA/AG^b^	85	19	u.a.	1.00
GG	5	0		u.a.
**rs713330**				
TT^b^	80	16	0.719	1.00
TC/CC	12	3		1.15 (0.34-3.95)
TT/TC^b^	90	19	u.a.	1.00
CC	2	0		u.a.
**rs11821102**				
GG^b^	76	18	0.297	1.00
GA/AA	15	1		0.33 (0.04-2.46)
GG/GA^b^	91	19	u.a.	1.00
AA	0	0		u.a.
**rs10836347**				
CC^b^	74	15	0.530	1.00
CT/TT	16	5		1.44 (0.52-3.95)
CC/CT^b^	90	19	0.182	1.00
TT	0	1		u.a.
**rs13347**				
CC^b^	38	7	0.627	1.00
CT/TT	55	13		1.21 (0.48-3.03)
CC/CT^b^	85	16	0.221	1.00
TT	8	4		2.08 (0.69-6.21)
**Clinical stage**				
stage I^b^	60	6	0.005^*^	1.00
≥ stage II	34	14		3.47 (1.33-9.04)
**Pathologic type**				
squamous cell carcinoma^b^	86	16	0.219	1.00
adenocarcinoma	8	4		2.10 (0.70-6.29)
**Cell grading**				
well (grade 1)^b^	14	3	1.000	1.00
moderate & poor (grades 2/3)	80	17		1.03 (0.30-3.52)
**Stromal invasion depth**				
≤ 10 mm^b^	54	4	0.003^*^	1.00
> 10 mm	38	15		4.56 (1.51-13.75)
Tumor diameter				
≤ 4 cm^b^	56	5	0.004^*^	1.00
> 4 cm	37	15		4.03 (1.46-11.09)
**Parametrium**				
no invasion^b^	65	7	0.003^*^	1.00
invasion	28	13		3.42 (1.36-8.57)
**Vagina**				
no invasion^b^	61	10	0.191	1.00
invasion	32	10		1.88 (0.78-4.51)
**Pelvic lymph node**				
no metastasis^b.^	77	5	<0.001^*^	1.00
metastasis	16	15		10.04 (3.64-27.70)

Statistical analyses: Kaplan-Meier curve model ^*^p<0.05 ^a^Clinicopathological data of some cases could not be obtained from the patients with cervical invasive cancer because of incomplete records of medical chart. ^b^As a reference. ^c^Only death event was considered, time interval was not included to calculate. ^d^HR, hazard ratio, time interval was considered; 95% CI, 95% confidence interval for CD44 genetic polymorphisms rs1425802, rs187115, rs713330, rs11821102, rs10836347 and rs13347 as well as clinicopathological variables, compared to their respective controls. Survival: +, survival, -, mortality; u.a., unavailable

**Table 5:** Multivariate analysis of genetic variants of CD44 and clinicopathological parameters for 5-year survival in cervical cancer patients

	5-year survival rate	
Parameters	*p* value	HR & 95% CI^b^
**CD44 genetic polymorphisms**		
**rs187115** AG/GG vs. AA^b^	0.014^*^	3.30 (1.28-8.49)
**Clinicopathological characteristics**		
**Pelvic lymph node** metastasis vs. no metastasis^a^	0.002^*^	8.57 (2.14-34.38)

Statistical analyses: Cox proportional hazard model ^*^p<0.05 ^a^As a comparison reference ^b^HR, hazard ratio and 95% CI, 95% confidence interval for significant univariate parameters CD44 genetic polymorphism rs187115 and clinicopathological characteristics, as compared to their respective controls. u.a., unavailable.
